# Evaluation of Targeted Next-Generation Sequencing for Detection of Bovine Pathogens in Clinical Samples

**DOI:** 10.1128/JCM.00399-18

**Published:** 2018-06-25

**Authors:** Eman Anis, Ian K. Hawkins, Marcia R. S. Ilha, Moges W. Woldemeskel, Jeremiah T. Saliki, Rebecca P. Wilkes

**Affiliations:** a Tifton Veterinary Diagnostic and Investigational Laboratory, College of Veterinary Medicine, University of Georgia, Tifton, Georgia, USA; b Department of Virology, Faculty of Veterinary Medicine, University of Sadat, Sadat City, Egypt; c Athens Veterinary Diagnostic Laboratory, College of Veterinary Medicine, University of Georgia, Athens, Georgia, USA; Medical College of Wisconsin

**Keywords:** bovine infectious diseases, diagnostic tools, targeted next-generation sequencing

## Abstract

The laboratory diagnosis of infectious diseases, especially those caused by mixed infections, is challenging. Routinely, it requires submission of multiple samples to separate laboratories. Advances in next-generation sequencing (NGS) have provided the opportunity for development of a comprehensive method to identify infectious agents. This study describes the use of target-specific primers for PCR-mediated amplification with the NGS technology in which pathogen genomic regions of interest are enriched and selectively sequenced from clinical samples. In the study, 198 primers were designed to target 43 common bovine and small-ruminant bacterial, fungal, viral, and parasitic pathogens, and a bioinformatics tool was specifically constructed for the detection of targeted pathogens. The primers were confirmed to detect the intended pathogens by testing reference strains and isolates. The method was then validated using 60 clinical samples (including tissues, feces, and milk) that were also tested with other routine diagnostic techniques. The detection limits of the targeted NGS method were evaluated using 10 representative pathogens that were also tested by quantitative PCR (qPCR), and the NGS method was able to detect the organisms from samples with qPCR threshold cycle (*C_T_*) values in the 30s. The method was successful for the detection of multiple pathogens in the clinical samples, including some additional pathogens missed by the routine techniques because the specific tests needed for the particular organisms were not performed. The results demonstrate the feasibility of the approach and indicate that it is possible to incorporate NGS as a diagnostic tool in a cost-effective manner into a veterinary diagnostic laboratory.

## INTRODUCTION

Infectious diseases continue to cause problems for the cattle industry worldwide, and their effective control is crucial for animal health and welfare (1). The first line to control an infectious disease problem is to apply rapid, sensitive, and accurate diagnostics. Accurate infectious disease diagnostic methods are important for determining disease prevalence and for control of infectious diseases to enhance biosecurity. Having rapid and sensitive/specific diagnostic methods provides clinicians with information to make better pretreatment clinical and management decisions. This not only reduces the money, labor, and drugs spent on ineffective treatments, it can also reduce the suffering of affected animals by electing to cull or euthanize them (2).

PCR has become the gold standard test for detection of many viral, fungal, parasitic, and bacterial pathogens, surpassing virus isolation and bacterial culture methods in sensitivity (3, 4). However, while PCR assays are highly sensitive and specific, their use can be costly, especially when testing for multiple pathogens (5). Routinely, this is done by using many single PCRs or some multiplex PCRs. Multiplex PCR reduces costs, but the method is limited to a small number of pathogens that can be detected per test, potentially at the expense of sensitivity for each pathogen (6). As new technologies have become available, there is potential for development of improved diagnostic tests to PCR. One such technology is next-generation sequencing (NGS).

Deep (metagenomic) NGS has been applied to outbreak monitoring and in the discovery of new viruses that elude conventional tests (7). The technology can randomly amplify and detect all the pathogens that may be present in a sample, enabling universal unbiased pathogen detection. Unfortunately, this random amplification results in the amplification of all the nucleic acids, including host nucleic acids, which are more abundant than the pathogen nucleic acids. Therefore, millions of reads need to be analyzed to identify the pathogen(s) of interest. This drawback is one of the major hurdles that has delayed the implementation of the technology in the diagnostic laboratory because of the number of reagents that have to be used for deep sequencing and the need for curated databases to accurately evaluate such large data sets (8, 9). An alternative to metagenomic sequencing is targeted NGS. Targeted NGS refers to the selective capture or amplification of specific genomic regions of interest prior to massive parallel sequencing. Compared to metagenomic sequencing, the possibility of targeted sequencing discovering new pathogens is limited. However, selectively sequencing pathogens of interest provides better sensitivity, better specificity, ease of downstream analysis, and lower cost by allowing more samples to be tested in one run (10). Targeted NGS has been applied successfully in cancer diagnostics (11–13). All of these advantages suggest that targeted NGS can be used for syndromic testing by providing a comprehensive diagnostic assay for the detection of known, clinically relevant pathogens from a variety of specimens, particularly for cases that present nonspecific disease signs that may be associated with multiple infectious agents. Therefore, in this study, we tested 60 clinical samples (including tissues, feces, and milk) with the targeted NGS assay, along with routine diagnostic methods, to evaluate the feasibility of applying a targeted NGS technique to syndromic testing (for mastitis, enteritis, and respiratory and reproductive disease) in a clinical molecular diagnostic laboratory.

## MATERIALS AND METHODS

### Design of amplicon panel primers.

We designed and evaluated 198 primers (see Table S1 in the supplemental material) divided into two primer pools that target most of the common bovine and small-ruminant bacterial, fungal, viral, and parasitic pathogens (43 pathogens) (Table 1). These primers were designed using the Ion Ampliseq Designer (Ion Torrent; Thermo Fisher Scientific, Waltham, MA), a primer design tool to create custom panels for targeted sequencing, and changes were made to the design with the assistance of the White Glove Team (Ion Torrent; Thermo Fisher Scientific). Because variations in targeted organisms could cause individual primers to fail, we designed multiple primers for most of the targeted organisms to provide redundancy.

**TABLE 1:** Validated isolates and reference strains and their sources

Pathogen	Source^*a*^
Bovine viral diarrhea virus (strain Singer, type 1a)	A
Bovine viral diarrhea virus (strain 125, genotype 2)	A
Bovine herpesvirus type 1 (strain Colorado)	A
Bovine herpesvirus type 4 (DN-599)	A
Bovine coronavirus (strain Nebraska)	A
Bovine respiratory syncytial virus (strain A51908)	A
Influenza virus type D	B
Parainfluenza virus type 3 (strain SF-4)	A
Rotavirus A (strain Nebraska)	A
Bluetongue virus (serotype 10 tested)	A
Adenovirus type 3	C
Mannheimia haemolytica	C
Trueperella pyogenes	C
Bibersteinia trehalosi	C
Histophilus somni	C
Mycoplasma spp. (Mycoplasma bovis tested)	C
Escherichia coli strains that possess Shiga toxin 1 (*stx1*), intimin (*eae*), alpha hemolysin (*hlyA*), cytotoxic necrotizing factor (*cnf1* and *cnf2*), enterotoxin (*STa*), fimbrial (*k99*), and *F41* virulence factor genes	D
Clostridium perfringens strains that possess α, β, β-2, enterotoxin (CPE), ε, and ι toxin genes;	C
Mycobacterium avium subsp. paratuberculosis (MAP)	C
Salmonella spp.	C
Campylobacter fetus subspecies (Campylobacter fetus subsp. *fetus* and Campylobacter fetus subsp. *venerealis*)	E
Listeria monocytogenes	C
Brucella spp. (B. canis tested)	C
Leptospira spp. (serovar Pomona tested)	A
Ureaplasma spp. (U. parvum tested)	F
Staphylococcus aureus	C
Coagulase-negative Staphylococcus (S. epidermidis tested)	C
Streptococcus agalactiae	C
Streptococcus dysgalactiae	C
Streptococcus uberis	C
Nocardia spp. (*N. nova* tested)	C
Pseudomonas spp. (P. aeruginosa tested)	C
Klebsiella spp. (K. pneumoniae tested)	C
Chlamydia spp. (C. felis tested)	C
Anaplasma marginale	C
Neospora caninum	G
Toxoplasma gondii	G
Tritrichomonas fetus	F
Cryptosporidium spp. (C. parvum tested)	C
Giardia intestinalis	C
Prototheca spp. (*P. zopfii* tested)	E
Aspergillus spp. (A. fumigatus tested)	C
Fusarium spp.	C

a A, USDA National Veterinary Services Laboratory; B, kindly provided by Henry Wan, Mississippi State University; C, detected in a diagnostic sample and identified/isolated in the TVDIL according to a validated protocol; D, E. coli Reference Center, Pennsylvania State College of Agriculture Sciences; E, validated reference kindly provided by Amy Swinford, Texas Veterinary Medical Diagnostic Laboratory; F, American Type Culture Collection, Manassas, VA; G, validated reference kindly provided by Chunlei Su and Rick Gerhold, University of Tennessee. TVDIL is accredited by the American Association of Veterinary Laboratory Diagnosticians (requirements are based on the ISO/IEC 17025 2005 standard *General Requirements for the Competence of Testing and Calibration Laboratories*).

We initially evaluated the panel by sequencing reference strains and known isolates of bacteria, parasites, fungi, and viruses to determine the specificity of the method and to identify targets with poor or no sequencing coverage. The primer pools were redesigned to contain alternate primer sets for poorly sequencing targets, and the primer pools were then reevaluated.

### Nucleic acid extraction.

Total nucleic acid was isolated using the DNeasy blood and tissue kit (Qiagen, Valencia, CA), which has been shown to extract both DNA and RNA. A modification of the animal tissue protocol was employed, adding the following step: following mechanical disruption of the tissue, the sample supernatant was transferred to a bead tube (Thermo Fisher Scientific) and vortexed for 10 min, followed by centrifugation at ≥6,000 × *g* (8,000 rpm) for 1 to 2 min. Then, the manufacturer's protocol was followed for purification of the nucleic acid through the column. For fecal/enteric clinical samples, an additional step was added: following resuspension of the fecal sample in phosphate-buffered saline (PBS) or mechanical disruption of the tissue (intestine), the sample supernatant was exposed to two freeze/thaw cycles (−80°C followed by heating at 70°C). Then, the sample supernatant was transferred to a bead tube, and the extraction process was continued as described above. For milk, a pellet was obtained from at least 5 ml of milk samples from individual animals or 30 ml from a bulk tank. Then, the cell pellet was suspended in 500 μl PBS and transferred to a bead tube, and the extraction process was continued.

### Library preparation and next-generation sequencing.

The sample DNA/RNA concentration was measured using a Tapestation 4200 in combination with the Agilent high-sensitivity D1000 screen tape assay kit (Agilent Technologies, Santa Clara, CA). Automated library preparation, template preparation, and chip loading were performed using the Ion Chef instrument. Automated preparation minimizes sample handling and lowers the chance of contamination, as well as providing reproducible chip loading. For library construction, up to 70 ng of DNA/RNA was amplified using the designed panel (Ampliseq; Thermo Fisher Scientific) and the Ion Ampliseq kit for Chef DL8, according to the manufacturer's protocol, with less than 15 min hands-on time. The kit allowed the preparation of 8 barcoded Ion Ampliseq libraries per Ion Chef run (8 different clinical cases) in about 7 h, depending on the number of cycles used for target amplification (21 cycles were used). Then, 50 pM of the 8 mixed libraries was templated and loaded on an Ion 314 chip using the Ion Chef instrument with the Ion PGM Hi-Q Chef kit (Thermo Fisher Scientific), according to the manufacturer's instructions, in about 12 h (overnight). Briefly, the prepared library was clonally amplified on the Ion Chef system by emulsion PCR of library molecules captured on beads. The Ion Chef system performed all the template preparation steps, including creating the emulsion mixture, performing the PCR, carrying out the post-PCR purifications, and finally loading the purified templated beads onto the Ion 314 chips. Finally, the libraries were sequenced on an Ion Torrent pErsonal genome machine (PGM) (Thermo Fisher Scientific) sequencer with the Ion PGM Hi-Q sequencing kit (Thermo Fisher Scientific), according to the manufacturer's instructions, in about 4 h, with 15 min hands-on time. A negative extraction control (NEC) was used to detect any contamination that might occur during the extraction and/or the library preparation process. This automated workflow generated the results in 2 to 3 days from sample receipt.

### Data analysis.

A reference file containing the sequences of the targeted pathogens and a bed file based on the locations of the designed primers were constructed and uploaded to the Ion Torrent suite software (Thermo Fisher Scientific). The files were used for initial data analysis with the Torrent suite software. The Torrent suite software provides the tools that convert raw sequence data to informative results, including optimized signal processing, base calling, and sequence alignment in Bam file format. The generated Bam files were downloaded and evaluated with Geneious software (version 9.1.2; Biomatters). Finally, pathogen identifications were confirmed with NCBI BLAST. BLAST (E) values of less than 1 were considered acceptable.

### Assay analytical performance.

Both analytical sensitivity (LOD) and specificity were determined to test the analytical performance of the assay. The relative analytical sensitivity was determined by testing relatively known quantities (based on qPCR/qRT-PCR results and *C_T_* values) of DNA from representatives of the viral, bacterial, fungal, and parasitic pathogen groups, as well as viral RNA. Real-time PCR/RT-PCR assays were performed at the Tifton Veterinary Diagnostic and Investigational Laboratory (TVDIL), the Athens Veterinary Diagnostic Laboratory (AVDL), and the University of Tennessee Veterinary Medical Center Clinical Virology and Immunology Laboratories, with laboratory-validated procedures. Typically, for real-time PCR testing, higher *C_T_* values (above 35) are considered suspect, as they may represent only amplification/fluorescence artifacts or cross contamination (29).

Analytical specificity testing was performed to determine the ability of the assay to detect the intended targets without being affected by specimen-related conditions or cross-reactivity/interference of the host nucleic acid. The assay was evaluated by testing validated isolates/reference strains of all the pathogens (bacteria, parasites, fungi, and viruses) that can be detected using the panel (Table 1). To better mimic clinical samples, a sample known to contain one pathogen based on previous testing was spiked with equal amounts of three or more of the tested isolates.

### Assay clinical evaluation and statistical analysis.

Determination of the clinical sensitivity (PPA) and specificity (NPA) of an assay requires the evaluation of every detected pathogen in every clinical case and comparing the sensitivity/specificity with those of reference standards. Given the number of pathogens that the bovine-targeted NGS assay can detect, determining the exact PPA and NPA for all the individual targets is cost prohibitive for any veterinary diagnostic laboratory. To overcome this challenge, 60 different clinical cases (57 bovine and 3 caprine) that were submitted to TVDIL and AVDL, University of Georgia, for diagnostic testing from 2015 to 2017 were included in a comparative study. The samples tested were feces (enteritis cases), milk (mastitis cases), and pooled tissue (a portion of the affected tissue was usually used, for example, lung or trachea for respiratory cases; the affected section of the intestine for enteric cases; and placenta, fetal lung, liver, kidney, brain, or stomach contents in cases of abortion). Each case was tested using the designed targeted NGS assay, along with routine laboratory diagnostic methods (reference methods). The routine diagnostic tests included bacterial cultures, DFAT, enzyme-linked immunosorbent assay (ELISA), PCR, qRT-PCR, and qPCR. When there was a discordant result between the targeted NGS and one of the routine diagnostic assays, the sample was examined by another, more sensitive/specific (generally PCR) diagnostic assay. The clinical cases included 16 mastitis cases, 15 respiratory cases, 20 enteric cases (2 caprine and 18 bovine), and 9 reproductive/abortion cases (1 caprine and 8 bovine). The relative PPA and NPA were assessed with respect to conventional/reference methods as a group (17). Also, the overall agreement and Cohen's kappa (the standard agreement coefficient) were assessed by looking across all the pathogens that can be detected by the new assay relative to conventional/reference methods as a group. Relative PPA and NPA calculations were performed for a few individual organisms within the syndromes tested, and those results are also included.

## RESULTS

The new assay was able to specifically detect/sequence the genes of interest of all the isolates of the bacteria, fungi, parasites, and viruses that were included in the panel. The relative limits of detection (LOD) of the various group (bacterial/viral/parasitic DNA and viral RNA) representatives were threshold cycle (*C_T_*) values of 30 to 38 (Table 2).

**TABLE 2:**
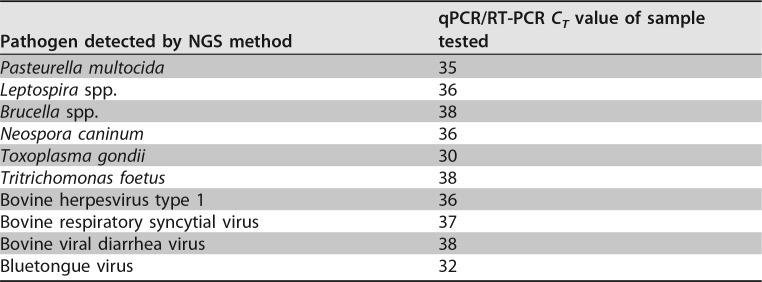
Limits of detection

There were concordant results among 11 out of 16 milk samples for which the targeted NGS assay and the routine diagnostic test were compared. In 2 milk samples, the targeted NGS assay was able to detect additional pathogens that were not detected by culture (Table 3). Other discrepancies were related to culture detecting organisms for which primers were not included in the NGS panel.

**TABLE 3:**
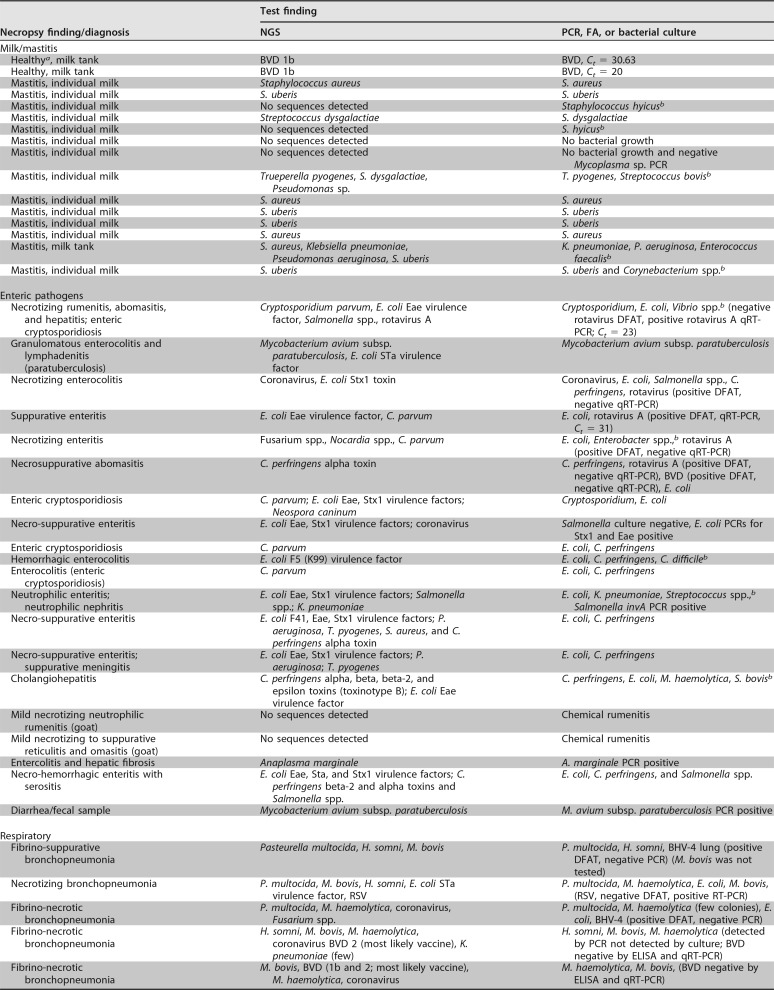

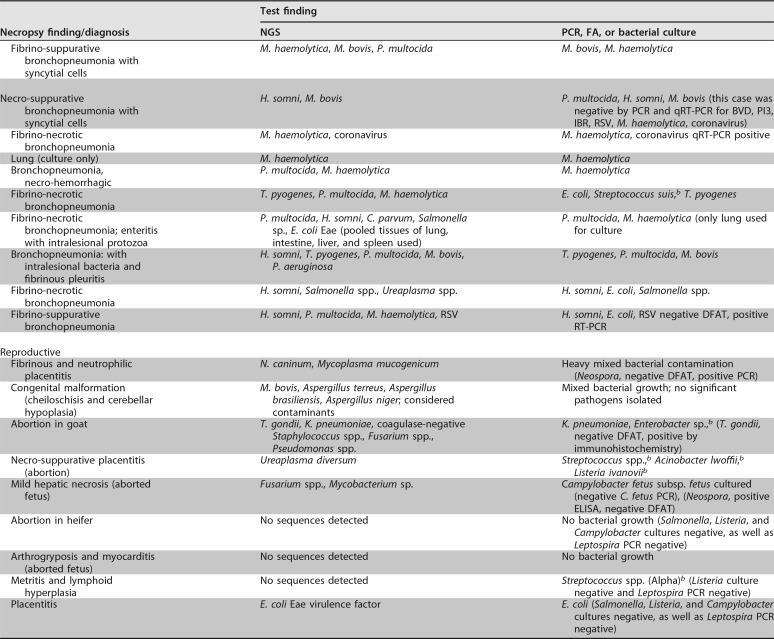
Clinical case comparative study results

^*a*^Milk sample was collected from a healthy animal as part of a study that was done in the TVDIL.

^*b*^Primers for this pathogen were not included in the panel.

Among the 20 enteric cases that were examined, Escherichia coli was cultured from 3 cases and Clostridium perfringens was cultured from 4 cases in which neither organism was detected by targeted NGS (Table 3). Also, the NGS assay was not able to detect rotavirus A in 4 enteric cases that were rotavirus A positive by direct fluorescent antibody test (DFAT). Examination of these 4 cases by a commercial rotavirus quantitative reverse transcription (qRT)-PCR (LSI VetMax Triplex; Thermo Fisher Scientific) confirmed the detection of rotavirus in only 1 case, with a *C_T_* value of 31 (Table 3). In one of the additional enteric cases, rotavirus was not detected by DFAT, while the targeted NGS assay and qRT-PCR were able to detect the rotavirus (*C_T_* = 23).

In 2 out of 9 reproductive cases, the targeted NGS assay was able to detect Toxoplasma gondii and Neospora caninum, which were missed by DFAT (Table 3). In one of the reproductive cases, the targeted NGS assay was not able to detect Campylobacter fetus subsp. *fetus*, which was detected in moderate numbers using a Campylobacter-specific culture technique, but interestingly, this sample also tested negative for the bacterium by quantitative PCR (qPCR) (Table 3).

Among the 15 respiratory cases included in the comparative study, multiple pathogens were detected in each case by both targeted NGS and bacterial culture (Table 3). The targeted NGS assay was able to detect bovine viral diarrhea (BVD) virus vaccine strains in two respiratory cases. It was also able to detect respiratory syncytial virus (RSV) in 2 respiratory cases in which it was missed by DFAT but confirmed by RT-PCR. The targeted NGS assay was not able to detect bovine herpesvirus 4 (BHV-4) in two respiratory cases in which it was detected by DFAT. However, the DFAT results were shown to be false positive, based on negative results obtained for the samples when tested by a herpesvirus consensus PCR (14).

Based on the results of the comparison between NGS and the reference diagnostic tests, the positive percent agreement (PPA) and negative percent agreement (NPA) of the designed targeted NGS assay were 86% and 81%, respectively. The overall agreement between the new assay and the routine methods used in this comparison was 85%, with kappa (K) equal to 0.64, i.e., substantial agreement (95% confidence interval [CI], 0.42 to 0.85), as shown in Table 4. Within the mastitis group, the PPA for Staphylococcus uberis was 100% and the NPA was 92%. In the enteritis group, the PPA for Cryptosporidium was 100% and the NPA was 88%. Within the enteric and respiratory syndromes, for cases of E. coli infection, the PPA was 70% and the NPA was 100%, and for C. perfringens, the PPA was 56% and the NPA was 100%. For respiratory cases, the PPA for Mannheimia haemolytica was 78% and the NPA was 67%, and for Pasteurella multocida, the PPA was 71% and the NPA was 63%. The PPA for Histophilus somni was 100% and the NPA was 70%, and the PPA and NPA for Mycoplasma bovis were both 100%.

**TABLE 4:** Comparison of targeted NGS assay and conventional/reference methods

Targeted NGS panel^*a*^ result	No. with conventional/reference method result of:		
	Positive	Negative	Total
Positive	38	3	41
Negative	6	13	19
Total	44	16	60

a The new assay.

## DISCUSSION

Current tests for infectious disease diagnosis rely on culture, antigen detection, and PCR. These methods often have low sensitivity and/or specificity, long turnaround times, or limited scope. As a result, the etiologies of many infections remain unknown, and patients are treated empirically. This leads to missed opportunities for targeted treatment and the overuse of antibiotics. Recent advances in NGS have provided an opportunity for the development of new research and diagnostic techniques (15). This study showed the feasibility of the use of targeted NGS as a method for molecular detection of various types of pathogens in the same sample with a single test. The assay was able to detect pathogens that had high *C_T_* values, demonstrating the ability of the assay to detect the pathogens even if they were present at low levels. Evaluation of analytical sensitivity is usually done by testing the LOD with plasmids or *in vitro*-transcribed RNA. Considering the lack of availability of all the reference material needed to perform this type of testing and the prohibitive cost, we decided to limit this evaluation to relative LOD for group representatives of viral/bacterial/parasitic DNA and viral RNA. Though this is not best practice, the test is intended to detect organisms associated with clinical disease, so determining the absolute analytical sensitivity was considered less important than it would be if the assay was instead used for testing in which a very low limit of detection is needed, such as for foreign animal diseases or for biothreat agents. However, targeted NGS has been shown to be very sensitive and has been used for this type of testing. A targeted NGS approach was able to detect biothreat pathogens with as little as 10 copies/ml in a sample that contained multiple pathogens and was spiked with human genome. Though the sample used in that study did not reflect a true sample matrix, it showed the ability of targeted NGS to detect pathogens of interest in a complex matrix (16). In the current study, the targeted NGS assay was able to specifically detect the targeted pathogens, as well as to provide enough pathogen coverage over the host background from various sample types, effectively removing the hurdle created by the host background nucleic acid to the application of NGS as a routine clinical diagnostic test. The bovine targeted NGS panel was able to detect pathogens that gave a *C_T_* value as high as 38. While this *C_T_* value would normally be considered a suspect result with real-time PCR testing, the use of targeted NGS provides the sequencing data to allow confirmation of detection of such small amounts of target in the sample.

One of the limitations of the assay is that it is restricted to only those organisms targeted by the primer sets used, and this limitation was demonstrated with the milk samples that were tested. In some cases, the NGS assay was unable to detect organisms that were detected by culture because the primers for those organisms were not included in the panel, as they were not considered to be of interest. It is important to note the scalability of the assay. Additional primers can be added, up to a total of 24,000 targets per tube. Therefore, primers can be added as needed with the discovery of new pathogens of interest.

In this study, though the overall agreement between the new assay and the reference tests was high (nearly perfect agreement), the kappa value showed substantial reliability. The kappa statistic is commonly used to measure agreement between two tests. However, when a new assay is being compared to an imperfect reference, the PPA and NPA are more appropriate metrics of comparison than the overall agreement or the kappa statistic. The overall percent agreement does not differentiate between agreement on the positives and agreement on the negatives. Kappa statistics is sensitive to the distribution of the marginal totals. A substantial imbalance in the marginal totals, shown in Table 4, either vertically or horizontally, can result in high agreement but a lower kappa value (17), which is what was observed in this study. This type of comparison among the entire group of organisms detected is more suitable for a metagenomic-type test and over- or underrepresents the PPA and NPA for each individual target, as shown by evaluating a few organisms individually. However, it does give a general idea of the capability of the method. It is important to note that the number of cases for each individual organism was very small, and additional testing to evaluate all the discrepant results could not be performed for each individual case.

The failure of the targeted NGS method to detect rotavirus in an enteric case that was positive by a commercial rotavirus-specific qRT-PCR (*C_T_* = 31) was likely due to reduced sensitivity of the targeted NGS method compared to rotavirus-specific qRT-PCR. However, the high *C_T_* value of this case revealed a very small amount of viral RNA in the sample, suggesting that rotavirus was not necessarily a significant cause of the enteritis in this case; indeed, Cryptosporidium, which was also detected, appears to have been the primary cause of the clinical disease. Therefore, given the lack of clinical significance of the PCR findings, the reduced sensitivity to rotavirus in the NGS method is not a concern (18). The positive rotavirus DFAT results in 3 enteric cases that were negative by commercial rotavirus A-specific qRT-PCR indicate that DFAT is problematic as a diagnostic tool for rotavirus. Though DFAT provides rapid results, its diagnostic usefulness is limited by poor sensitivity and specificity, a cumbersome procedure, the quality of the antibody used, and subjective reading/interpretation of results (19, 20).

DFAT also falsely detected BHV-4 in two respiratory cases. Additionally, DFAT failed to detect RSV in 2 cases in which it was detected by the targeted bovine NGS panel. These results add to the body of evidence that the DFAT does not perform as well as newer diagnostic methods in detecting viruses.

NGS failed to detect E. coli and/or C. perfringens that was cultured by routine methods in several of the enteric cases, resulting in reduced PPA for both organisms. E. coli and C. perfringens are part of the normal intestinal flora and are commonly isolated from the feces/intestines of apparently healthy animals (21). Consequently, it is sometimes difficult to determine if these organisms are true pathogens based on culture results alone. To overcome this problem, the targeted NGS assay was designed to detect E. coli and C. perfringens virulence factors/toxins to discriminate commensals from pathogenic bacteria. Therefore, lack of detection of virulence factors or toxins in these specific cases suggests the culture of normal flora or perhaps the presence of virulence factors that were not detected by the assay because the specific primer set was not included in the panel. Based on detection of other pathogens in these cases that could account for the observed lesions, it was concluded that culture of normal flora was more likely (Table 3).

Among the 15 respiratory cases, the targeted NGS assay was able to detect BVD virus vaccine strains in 2 cases that were not detected by DFAT and qRT-PCR. This result demonstrates the ability of the assay to type the BVD present in the samples. Design of primers to include regions for strain determination or to distinguish between vaccine strains and wild-type strains is an additional benefit of using targeted sequencing.

Bovine respiratory disease is a multifactorial disease in which multiple pathogens can infect the lung; it is not possible to determine the relative primary or secondary significance of the pathogens by culture or NGS methods. NGS may not have detected the pathogens that were present in the sample in very low loads and were outcompeted by the other pathogens that were abundant in the cases. This resulted in variability in the PPA and NPA among the organisms detected. Generally, the detection of multiple pathogens in a clinical sample by either targeted NGS or conventional diagnostic methods should be evaluated in light of the clinical disease and pathological findings. Detection of a bacterium or virus in a sample does not necessarily discriminate the true pathogen from an innocent bystander or a component of the normal flora. Organism quantity in a sample is helpful for determining clinical significance, and the targeted NGS method can give a general estimate of the pathogen load via the read numbers, but this is not as absolute as quantitative real-time PCR. Additionally, like all molecular methods, NGS does not discriminate living from dead organisms.

Among the 9 reproductive cases examined, the targeted NGS assay was unable to detect C. fetus subsp. *fetus* in one case in which it was cultured, but the organism was also not detectable by qPCR. The routine method for the diagnosis of C. fetus is the culture and identification of the causative organism. The identification of isolates is problematic due to the limited biochemical activity of the bacteria. Therefore, concerns have been raised regarding the sensitivity of the culture and the identification techniques used (22, 23). For the particular sample discussed here, the pathologist determined C. fetus to be the most likely cause of the abortion.

Though, the assay was able to detect multiple pathogens in caprine samples, more small-ruminant samples should be examined to validate the ability of the assay to detect major infectious pathogens in clinical cases caused by these species.

In addition to pathogen identification, NGS can predict the phenotypic antimicrobial resistance by targeting known genetic determinants of antimicrobial resistance (24, 25). However, the accuracy of phenotypic susceptibility prediction via NGS is still under investigation (26–28). Correlation between genotypic data and a clinical phenotype is complicated and includes various mutations that cause similar phenotypic changes, so antimicrobial susceptibility testing (AST) was not included in the targeted NGS assay presented here. Therefore, traditional AST is still essential, and it is recommended to have an initial bacterial culture plate from samples that will be tested by the targeted NGS bovine assay in case AST is required.

These results demonstrate that, overall, the targeted NGS bovine assay is sensitive and specific in detecting the major bovine pathogens in clinical cases and therefore can be incorporated into the veterinary laboratory diagnostic method in a cost-effective manner. Moreover, the workflow is straightforward, with a turnaround time of 2 to 3 days from receipt of the sample. The cost per sample is approximately $200 and is comparable to that of most commercial multipathogen molecular assays due to the reduced number of sequences needed to achieve reliable results. The development of nucleic acid-based tests, including targeted NGS, provides information complementary to that from conventional culture-based tests and serology. Multiplex molecular applications are rapidly evolving and will eventually have a significant impact on diagnostic laboratory turnaround time.

In conclusion, targeted NGS offers the scalability, speed, reproducibility, and resolution to detect targeted genes of interest. Multiple pathogens can be detected across many samples in parallel, saving time and reducing costs associated with running multiple separate assays. Further, targeted gene sequencing produces a smaller, more manageable data set than whole-genome sequencing, making analysis easier (16). The use of targeted NGS for infectious disease diagnosis will provide accurate and rapid identification of pathogens so that the most appropriate and effective treatment can be applied quickly.

## Supplementary Material

Supplemental material
