# Supplementary material for: Evaluation of Targeted Next-Generation Sequencing for Detection of Bovine Pathogens in Clinical Samples
Source: J Clin Microbiol. 2018 Jun 25;56(7):e00399-18. doi: 10.1128/JCM.00399-18 (PMC6018347; doi:10.1128/JCM.00399-18)
Supplement: Supplemental material [file supp_56_7_e00399-18__index.html]

Evaluation of Targeted Next-Generation Sequencing for Detection of Bovine Pathogens in Clinical Samples — Supplemental material 

# Evaluation of Targeted Next-Generation Sequencing for Detection of Bovine Pathogens in Clinical Samples

## Supplemental material

- Supplemental file 1 -

  Table S1 (Bovine targeted NGS primers)

  XLS, 117K
